# Functional Connectivity in MRI Is Driven by Spontaneous BOLD Events

**DOI:** 10.1371/journal.pone.0124577

**Published:** 2015-04-29

**Authors:** Thomas W. Allan, Susan T. Francis, Cesar Caballero-Gaudes, Peter G. Morris, Elizabeth B. Liddle, Peter F. Liddle, Matthew J. Brookes, Penny A. Gowland

**Affiliations:** 1 Sir Peter Mansfield Imagaing Centre, School of Physics and Astronomy, University of Nottingham, University Park, Nottingham, United Kingdom; 2 Institute of Mental Health, University of Nottingham, Jubilee Campus, Nottingham, United Kingdom; 3 Basque Center on Cognition, Brain and Language, Donostia-San Sebastian, Spain; University Of Cambridge, UNITED KINGDOM

## Abstract

Functional brain signals are frequently decomposed into a relatively small set of large scale, distributed cortical networks that are associated with different cognitive functions. It is generally assumed that the connectivity of these networks is static in time and constant over the whole network, although there is increasing evidence that this view is too simplistic. This work proposes novel techniques to investigate the contribution of spontaneous BOLD events to the temporal dynamics of functional connectivity as assessed by ultra-high field functional magnetic resonance imaging (fMRI). The results show that: 1) spontaneous events in recognised brain networks contribute significantly to network connectivity estimates; 2) these spontaneous events do not necessarily involve whole networks or nodes, but clusters of voxels which act in concert, forming transiently synchronising sub-networks and 3) a task can significantly alter the number of localised spontaneous events that are detected within a single network. These findings support the notion that spontaneous events are the main driver of the large scale networks that are commonly detected by seed-based correlation and ICA. Furthermore, we found that large scale networks are manifestations of smaller, transiently synchronising sub-networks acting dynamically in concert, corresponding to spontaneous events, and which do not necessarily involve all voxels within the network nodes oscillating in unison.

## Introduction

Functional magnetic resonance imaging (fMRI) signals are often decomposed into a few, large scale, distributed networks within which blood oxygenation level-dependent (BOLD) signals are highly temporally correlated [1]. These networks are associated with sensory action (e.g. the sensorimotor network) [1,2], cognition or attentional processes (e.g. the default mode network (DMN)) [3–9]. The major features of these networks are remarkably consistent between individuals and can be observed at ‘rest’ or in the task evoked state. This coordinated activity across large networks is thought to be key to healthy brain function and has been reported to be perturbed in several pathologies [10–12].

Most functional connectivity studies assume spatio-temporal stationarity in the coupling between network nodes. However some studies have begun to assess dynamic changes in functional connectivity using both fMRI [13–16] and electrophysiological imaging techniques [17–19], showing that the connectivity between the nodes of well-characterised networks fluctuates over time. This implies that it is necessary to investigate processes occurring at higher frequencies than those typically assessed in BOLD fMRI, to understand fully the nature of large-scale functional connectivity. Furthermore, typical functional connectivity analysis detects relatively large network nodes [4], but since it is known that highly focal regions within the brain are involved in specific tasks, it is unlikely that large brain regions will always behave in the same way. For instance functional MRI studies showing that highly specific brain regions are involved in particular tasks, e.g. the motor cortex is segregated into regions responsible for individual fingers [20] and the auditory cortex is organized tonotopically [21]. It is more probable that patterns of functional connectivity will be modulated by spontaneous events that involve transiently synchronised activity in multiple brain regions, and these would give rise to transient, synchronized, evoked responses occurring in subregions of nodes of the relevant network. We contend that the large scale networks of connectivity detected with fMRI emerge from the combination of multiple sub-networks that transiently synchronise during spontaneous events that occur when the brain is apparently otherwise ‘at rest’, performing a task or responding to a stimulus.

In order to detect the BOLD response associated with spontaneous events, a model free analysis approach is required (i.e. one for which the timing of the events is not known *a priori*). Paradigm free mapping (PFM) [22] is such a model free approach in that it detects spontaneous BOLD events in fMRI data without prior knowledge of their timing and only assumes that any BOLD event will follow a canonical haemodynamic response function (HRF) shape [15,22,23]. Once spontaneous events have been detected, the next challenge is to interpret their functional significance, and to look for coherent patterns of spontaneous activity across the brain. If each detected BOLD event could be associated with a consistent pattern of activation, then it would be easier to determine what type of spontaneous brain activity it was generated by. Temporal independent component analysis (tICA) is a tool which can be used to reveal such consistent, temporally independent patterns of brain activity, but its use in fMRI is limited due to the fact that the BOLD signal is generally sampled at a low rate so that there is only a small number of time points are available for analysis [24]. However this constraint can be overcome by reducing the number of temporally independent components within the data, which we can achieve by applying tICA to the sparse output of PFM. The combination of PFM and tICA enables us to reveal consistent networks associated with spontaneous brain activity, each with different and temporally independent functional signatures.

Using this methodology, we tested the hypotheses that: 1) spontaneous BOLD events are related to dynamic variations in network connectivity; 2) these events involve consistent, spatially-structured activity within and across nodes of distributed networks, but may not involve the whole network; 3) performance of a task can change the rate of detection of spontaneous events.

## Methods

The University of Nottingham ethics committee approved this research. Written informed consent was received from each subject in accordance with the ethics. Twelve subjects from our lab (6 male, 1 left hander, mean age 25 ± 2.5 years) took part in the study, and each performed two experiments: a ‘Motor’ and a ‘2-back’ experiment. Each subject was instructed to remain at rest with their eyes open for 0<t<300s, perform a task for 300<t<660s and rest again for 660<t<1020s. The motor task consisted of a continuous, unilateral right hand finger tapping, whilst a sequence of letters was presented for the two back task. This was repeated at 2s intervals and the subject was asked to respond with an index finger button press when the current letter matched the letter presented two previously (a 2-back match occurred every 10s on average with 36 +/- 1 targets in each experiment). Both tasks were effectively continuous and were aimed at modulating baseline brain activity between the three periods (rest/task/rest) of the paradigm, rather than generating event-like activations during the task.

A schematic diagram of the analysis workflow is shown in the supplementary information (S1 Fig). T2*-weighted gradient-echo EPI data were acquired using a 7T Philips Achieva MRI system to provide increased BOLD sensitivity (2x2x2mm^3^, 30 slices, TE/TR: 25/2000 ms, SENSE: 3, bandwidth: 28 Hz). Cardiac and respiratory data were recorded using a vector cardiogram and respiratory belt. Data were initially realigned and slice timing corrected using SPM5 (Wellcome Trust, http://www.fil.ion.ucl.ac.uk/spm/software/spm5/). Datasets were excluded if movement was greater than 1 voxel (none excluded). Subsequently, RETROICOR [25] was used to remove non-neuronal physiological noise. Data were then spatially smoothed (Gaussian kernel with FWHM 4 mm) using SPM5 and finally corrected for drift by removing up to and including third order fitted polynomials. Four minute blocks of data were extracted for further analysis (rest 1: 1 –240s; task: 361 –600s; rest 2: 781 –1020s) to ensure there was no contamination due to the haemodynamic response from the start or end of the task.

Individual subject data sets were analysed using the probabilistic spatial ICA algorithm implemented in FSL [26]. Thirty spatial components were extracted and the components corresponding to the Motor Network (MN), Fronto-Parietal Network (FPN) and the Default Mode Network (DMN) were visually identified in each subject by comparison to a standard template [12]. Only thirty components were required as we only intended to identify robust resting state networks to be used as masks [4,27,28]. The MN was defined as consisting of 2 nodes, one in the left and one in right motor cortex; the FPN comprised 4 nodes in the lateral parietal area and dorsolateral pre-frontal cortex in the left and right hemispheres; the DMN consisted of 4 nodes in the posterior cingulate cortex and precuneus bilaterally, medial pre-frontal cortices bilaterally and posterior inferior temporal gyrus along with lateral parietal regions in the left and right hemispheres. The resulting nodes selected are shown in S1 Fig. For each network, a similar seed node was defined in all subjects. This was in the left motor cortex for the MN as most subjects were right handed, the posterior cingulate cortex in the DMN as it is the most robust and reliable node in that network, and the lateral parietal in the FPN. For each of these seeds the average signal timecourse was calculated and used for subsequent seed based correlation analysis (see Table 1).

**Table 1:** Showing characteristics of different events in each network, averaged over all time periods and all subjects, the error indicates the inter subject standard deviation.

Network	Seed region	Comparison node	Rate of detection of Nodal Events (min ^-1^)	Percentage of Nodal Events classified as Coordinated Network Events	Percentage of voxels in nodes involved in a nodal event
Motor (MN)	Left primary motor area	Right primary motor area	2.1 ± 0.4	41 ± 12%	31 ± 3%
Left fronto-parietal (LFPN)	Left posterior parietal region	Left dorsolateral prefrontal cortex (lDLPRC)	2.7 ± 0.3	34 ± 5%	27 ± 2%
Right fronto-parietal (RFPN)	Right posterior parietal region	rDLPFC	2.8 ± 0.4	36 ± 4%	24 ± 2%
Default Mode (DMN)	Posterior cingulate cortex.	medial prefrontal cortex (MPFC)	5.1 ± 0.8	17 ± 3%	30 ± 2%

Next, to identify spontaneous events Paradigm Free Mapping (PFM) [22] was applied. In brief, PFM involves solving a regularized inverse problem that deconvolves the HRF from the voxel wise signals using the Dantzig Selector estimator [23], and thus estimates the neuronal-related signals generating the BOLD responses. After the deconvolution a linear model was fitted to the voxel timecourse that included the BOLD responses estimated by the Dantzig Selector and the rotation and translation parameters estimated during realignment. This step was performed to provide a less biased estimate of the amplitude of the detected BOLD responses and to account for the variability of the signal due to motion-related effects. Importantly, the deconvolution is done without knowing when the responses occur and it only assumes a shape for the haemodynamic response, in this case the canonical HRF (Friston et al., 1998). The regularization parameter of the Dantzig Selector is chosen based on the Bayesian information criterion which constrains the estimates to be sparse but provides high specificity and sensitivity for detecting the BOLD responses [23]. This yielded an Activation Time Series (ATS) for each voxel indicating the timing and amplitude of detected deconvolved events. The method has been described and evaluated in detail in several previous publications [15,22,23].

**Fig 1:**
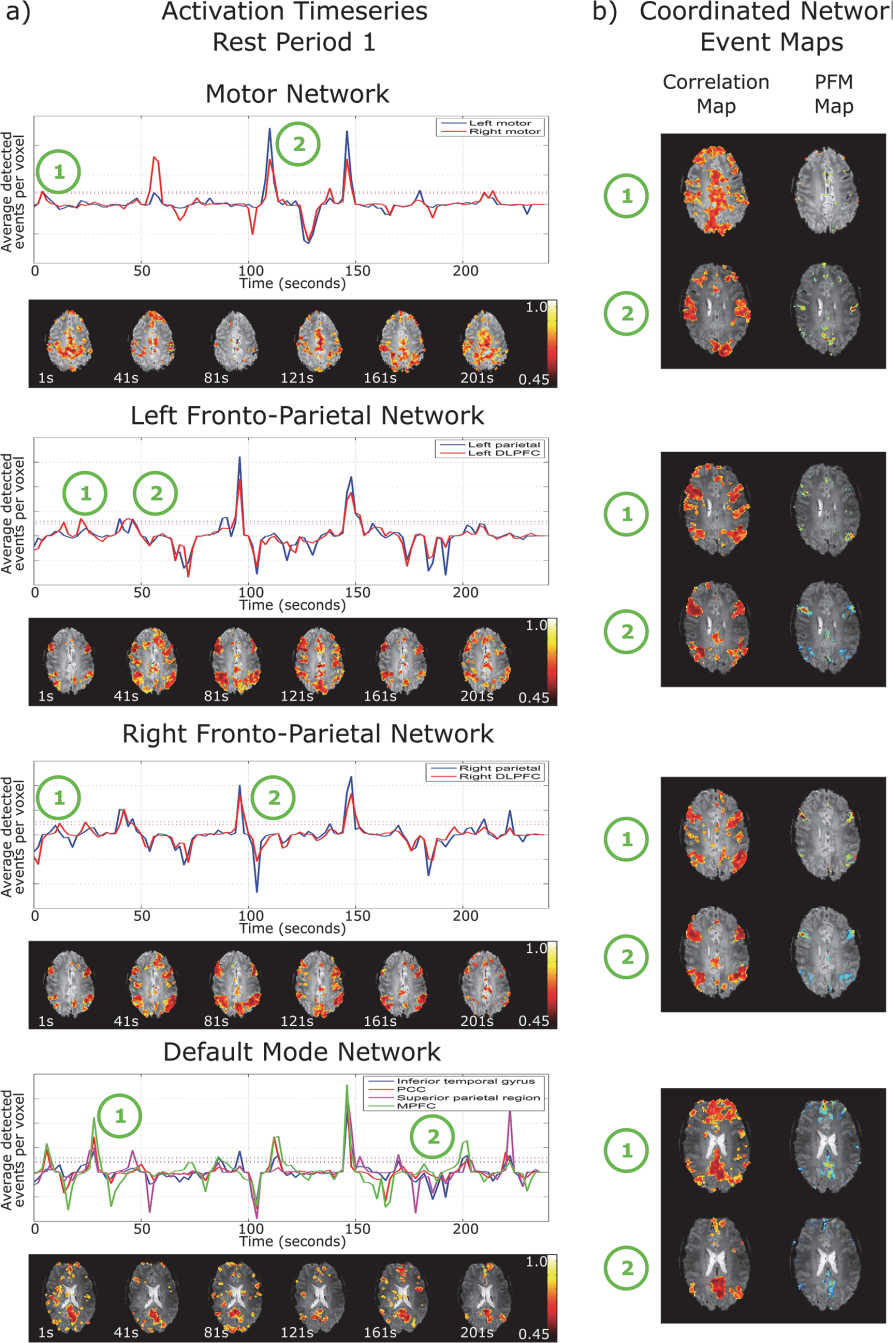
a) Nodal activation timeseries for the motor network, left and right fronto-parietal network and default mode network in rest period 1 from the motor data, for a single subject. The solid lines show the average number of voxels within the node defined as active by PFM at each time point, and the dotted lines depict one standard deviation from baseline. The correlation maps (below the activation timeseries) are shown for 30s time windows at 40s intervals, each window starting at the time indicated. These highlight the dynamic nature and changing structure of networks b) correlation maps at the time of a coordinated network event that show strong network structure and their corresponding paradigm free mapping activation map depicting the voxels that showed an event at this time.

Nodal ATSs were produced by summing the ATS over all voxels in each of the nodes of each network (Fig 1). Nodal Events were then defined as occurring when the nodal ATS signal exceeded 1 standard deviation above its mean representing an increase in spontaneous activity across a node (indicated by the dotted lines in Fig 1). A Coordinated Network Event was defined as occurring when all nodes of a network demonstrated a nodal event simultaneously. The Coordinated Network Events indicate spontaneous coordinated activity across the whole the network.

The Voxelwise Event Rate was assessed by calculating the number of spontaneous events found in any voxel (of any node) in the network per minute, normalised by the total number of voxels within the network. This was done for each network and each paradigm period to identify changes in rates of local (voxelwise) spontaneous activity caused by the introduction of a task.

To demonstrate how functional connectivity measures were affected by these detected events, for each network one Coordinated Network Event per subject was selected at random and the correlation map was computed for the 30s time window immediately following the onset of the event. The resulting correlation maps were transformed into MNI space using FLIRT [29] and were then averaged across all subjects for each network. A similar set of correlation maps was then created using 30s windows placed at times when no Coordinated Network Events had been detected (null periods) to compare periods of high and low spontaneous activity. These maps were then thresholded at a value of r = 0.45 that was determined to be the threshold for significance for a 30s window defined from a bootstrapping procedure. The bootstrapping procedure involved generating surrogate data from the actual fMRI data and randomising the phase of its Fourier transform so that the surrogate data still contains real signals and information. For each window length, a distribution of correlation values was computed using a seed in the grey matter and a threshold was taken at the 95^th^ percentile to determine a significant correlation for that window length.

To study the effect of events detected by PFM on the correlation analysis, sliding window correlation analysis [14,30,31] (2s steps, 10 to 240s window lengths) was performed between the average signal from a seed region and all the voxels in a comparison node of the network (Table 1) for each network. This was performed on three sets of data: before and after removal of all events detected by PFM [15] and after removal of only the Coordinated Network Events. Events found by PFM were removed from the raw data by subtracting the PFM-derived activation time series convolved with the canonical haemodynamic response function for each voxel. For each window length the fraction of sliding windows in which a significant correlation occurred (i.e. exceeding a bootstrap threshold estimated for each window length) was computed and averaged over voxels in the comparison ROI. This was called the Fraction of Significant Correlation (FSC) and allows comparison of the correlation observed at different window lengths to show how spontaneous events that are distributed across the network contribute to functional connectivity.

Finally, temporal ICA (tICA) was used to detect regions showing consistent, temporally independent patterns of activity related to events. tICA is generally not applied to fMRI data due to the limited number of time points acquired in a time series, but tICA can be applied to the sparse output of PFM since the number of temporal independent signals in the data is substantially reduced relative to the raw data (e.g. non-neuronal physiological noise is removed). Furthermore tICA was only applied within recognised functional networks to focus on a particular set of temporal components, whilst eliminating other confounding signals. For tICA analysis, data were masked using standard network masks for the MN, FPN and DMN [12]; standard masks were used at this stage since they were created independently of our data and analysis. For each network the voxelwise ATS outputs from PFM, were convolved with the canonical HRF from both rest periods and for both experiments, and were then concatenated and converted into a voxel by time matrix for each subject. PCA was applied to reduce the dimensionality of this matrix to 15 and tICA was then applied using the fastICA algorithm [32] to obtain 10 temporally independent components, each with its corresponding time course and spatial map. Maps of the tICA weights were produced showing the distribution of voxels in which temporally independent timecourses of activity occurred and illustrating sub-regions of the network which act in concert to form spontaneous events. These sub-networks are regions of the brain that transiently synchronise around an event and that exist within a larger distributed network

All statistical analyses were performed using 2-sided Wilcoxon signed rank tests since the data were not expected to be normally distributed given the variations in the numbers of events found between individuals.

## Results

The ATS, averaged over all voxels within separate nodes of each network, can be seen in Fig 1 for a single subject for the initial resting state block of the motor task. Peaks in the ATS exceeding one standard deviation above baseline indicate that BOLD events were simultaneously detected in different voxels within that node, and this was termed a Nodal Event. In some cases all nodes in a given network were found to be involved in a particular event and such events were classified as Coordinated Network Events. For comparison the acquired signal timecourses after RETROICOR preprocessing for the same subject and period can be found in the supplementary information (S2 Fig). The average correlation between the motion parameters and the ATS was r = -0.0085 implying that the events that have been detected do not relate to motion artefacts.

Sliding window seed correlation maps (30s window) computed at 40s time intervals from the pre-processed data are shown below each ATS in Fig 1A and illustrate the dynamic nature of functional connectivity, with large variations in network structure seen between time points. The correlation maps and PFM activation maps corresponding to two occasions when a Coordinated Network Event occurred for each network (at timepoints indicated in Fig 1A) show the expected structure of the relevant network at these times (Fig 1B). At time point t = 148s, there was a Coordinated Network Event in all four networks. Such events involving all networks simultaneously were termed global events. The 30s window correlation map computed at the time of a global event showed the entire grey matter to be significantly correlated with a seed.

Table 1 shows the rate of detection of Nodal Events, and the fraction of Nodal Events that were classified as Coordinated Network Events, averaged over all time periods and all subjects. It is notable that for Nodal Events only 28 ± 2% of voxels within the nodes showed a response as assessed by PFM [22], and this rose to only 34 ± 7% for Coordinated Network Events. Therefore even when all nodes of a network exhibited a BOLD response, the entire volume of each node was not generally involved. This suggests the existence of functional substructures within networks and nodes, which is expected given the highly focal, task specific network sub-regions that are detected in standard, task driven fMRI [33].

**Fig 2:**
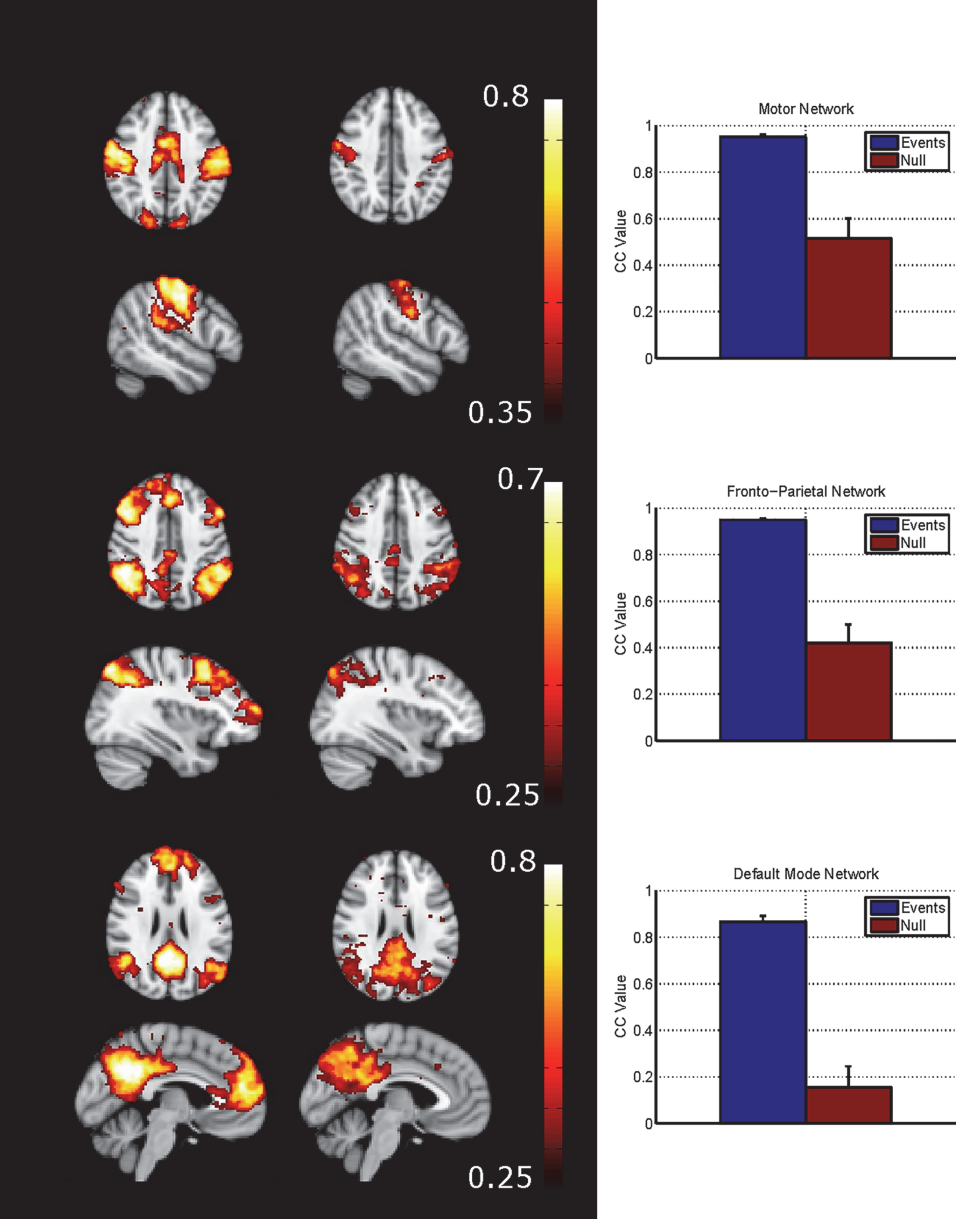
The cross subject average correlation maps following a coordinated network event (CNE- left) and for a null period (middle). The graphs (right) show the difference in connectivity at these periods with high connectivity following a CNE and low in a null period.

The subject-averaged correlation maps produced for 30 second windows following a Coordinated Network Event (for the three networks) demonstrate that full network structure can be defined from short periods of data (Fig 2, left column). However only limited network structure is found when combining correlation maps across subjects at a null period when no Coordinated Network Event had been detected (Fig 2, middle column). In the MN, the correlation map for the Coordinated Network Event period reveals the involvement of supplementary motor areas which are seen neither in the null periods, nor consistently in the sICA maps for each subject. Furthermore the Coordinated Network Event period maps of the FPN show clusters of significant correlation in areas of the DMN (e.g. medial prefrontal cortex) and *vice versa* (e.g. small clusters bilaterally in dorsolateral prefrontal cortex). The difference in average connectivity between the seed node and rest of the network for the period following a Coordinated Network Event is significantly higher than that during a null period (Fig 2, right column).

**Fig 3:**
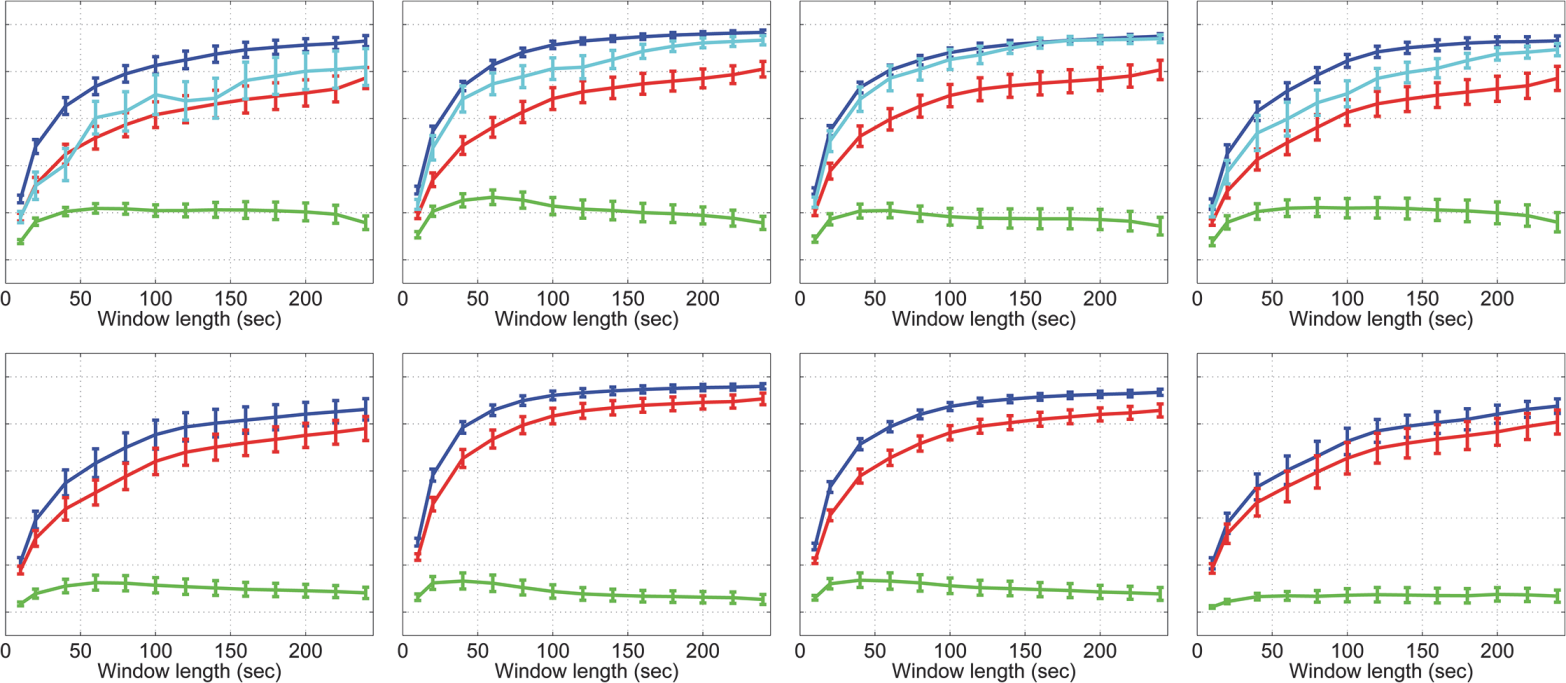
Fractional significant correlation scores for the 2-back data for rest period 1 (top row) and 2-back task period (bottom row).

The FSC score is significantly affected by the spontaneous events detected by PFM in such a way that the removal of these events reduced the temporal correlation between the seed node and rest of the network. Results are shown for the first resting state block and for the 2-back working memory task block (Fig 3). When events detected by PFM were removed there was a significant drop in the fraction of windows that showed significant connectivity (Wilcoxon sign rank test: p <0.001, uncorrected at all window lengths for non-task periods; p<0.025, uncorrected at all window lengths for task periods), indicating the contribution of spontaneous events to network connectivity. Considering the full 4 minute initial rest period, when all events were removed the average correlation coefficient dropped by 5–15% (significant across the subjects, p <0.002, uncorrected for all networks and periods). Coordinated Network Events contributed 29 ± 8% to this drop. Similar results were obtained for the motor task (shown in S4 Fig).

Before the data were corrected for spontaneous events the FSC score for the MN tended to be lower during the 2-back task (Fig 3B) than at rest (Fig 3A), particularly for short window lengths (significant for window lengths 20, 40, 60 and 120 s; Wilcoxon sign rank test p = 0.03, 0.03, 0.03 and 0.04 respectively, uncorrected). However, after removal of the spontaneous events the differences between task and rest in the MN were no longer significant, suggesting that such events were driving the difference in correlation. Interestingly, for the FPN there was no difference in the uncorrected data between task and rest, but after PFM correction, FSC values were higher in the task period than the rest period for all window lengths (see discussion). No increase in FSC scores was observed due to the motor task for any network.

**Fig 4:**
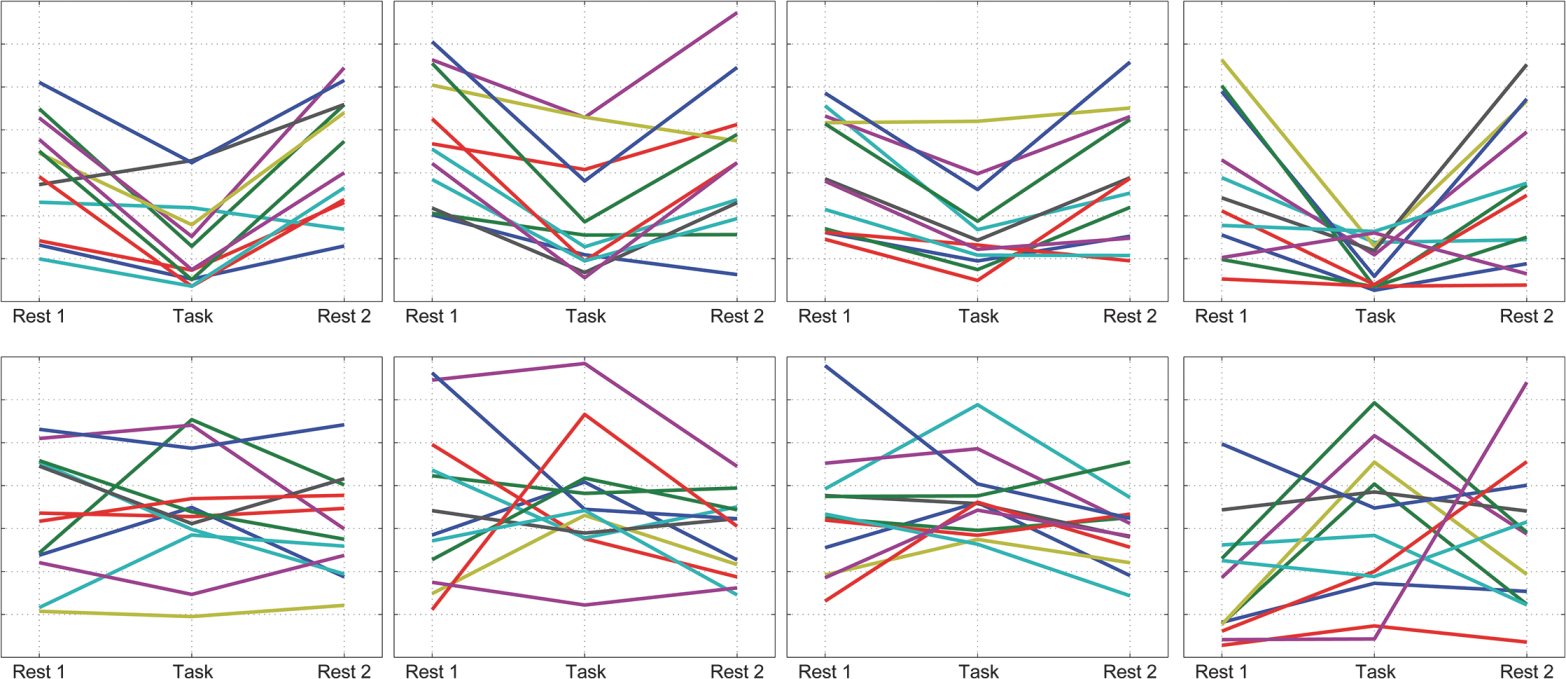
These graphs show the change in the average number of spontaneous events per voxel, per minute in each period. The 2-back data has a significant (Wilcoxon sign rank test p = 0.0025, 0.0005 and 0.001 for the MN, DAN and DMN respectively, uncorrected) decrease from rest to task period for all three networks. There is also a significant (Wilcoxon sign rank test p = 0.0425, uncorrected) increase in the number of events in the motor network for the motor data. The different colours represent different subjects

There was a significant change in the total number of spontaneous events found within individual voxels between task and rest periods, for all networks for the 2-back task, and the motor network for the motor task (Fig 4). However there was no significant change in the number of spontaneous Nodal Events or the number of Coordinated Network Events detected by PFM between the rest and task periods for any network. This suggests that a task influences the number of local spontaneous events but not the number of large scale coordinated events.

**Fig 5:**
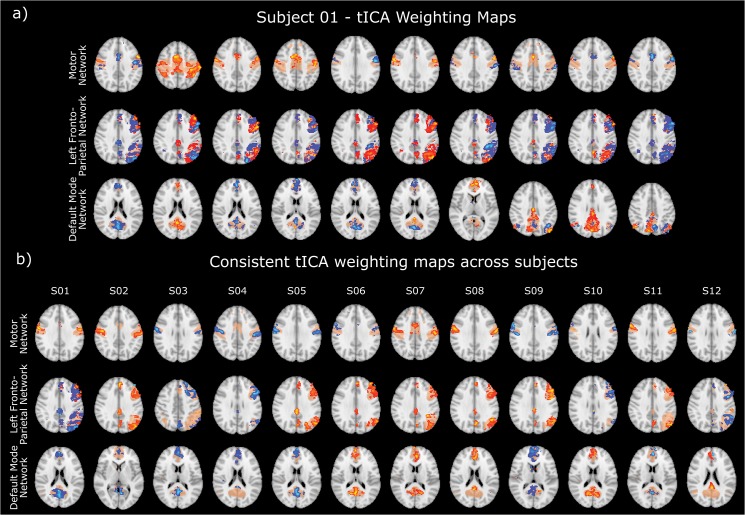
a) Ten tICA weighting maps for subject 1 for the three networks studied, showing sub-structures within each network mask (beige) and b) a single component tICA maps showing consistent patterns between different subjects for the MN, DAN left and DMN. The colour scale is normalised to unity.

Applying tICA to the output of the PFM analysis for voxels within the MN, left FPN and DMN highlights ‘transiently synchronising sub-networks’ that comprise multiple small clusters of voxels occupying only parts of the network nodes (Fig 5A shows the results for subject 1, where sub-networks 5 and 6 show bilateral motor whereas sub-network 2–4 shows bilateral pre-motor cortex). The results for other subject’s tICA maps for each network are shown in supplementary material S5–S15 Figs. The same transiently clustered voxels may contribute to multiple independent components, synchronised around spontaneous BOLD events occurring at different times; for example primary somatosensory area S1 is observed as part of most detected components and an overlap can also be observed between the sub-networks in components 5 and 6. The sub-networks show consistency across subjects as illustrated in Fig 5B which shows similar features in a single component between each subject for each network. Many other components showed similar consistency across subjects (see supplementary material S5–S15 Figs).

## Discussion

Functional connectivity is typically studied assuming that the temporal correlation between two brain regions is driven by low frequency oscillations. In this work, we used Paradigm Free Mapping [22,23] to detect spontaneous events in the nodes of brain networks that are frequently studied with functional connectivity MRI, and showed that these events contribute to network connectivity measured over short and long time-windows. Our findings demonstrate that these spontaneous events do not necessarily involve a whole network or node, but spatial clusters of voxels that act in concert forming transiently synchronising sub-networks. The results also show that introducing tasks may significantly alter the number of spontaneous events detected across a network and the frequency at which short term correlation (< 60s) occurs, even when no significant change can be detected in correlation calculated over an extended time period. These findings clearly indicate the dynamic and non-stationary behaviour of functional brain connections as measured with BOLD fMRI data.

The term ‘resting state’ is used to describe the situation when a subject is asked to do nothing, but clearly in this state the brain will be continuously performing tasks involving external stimuli, internal thoughts or movement [34], and likewise these spontaneous events will also occur in the task-evoked state. If the temporal sequence of spontaneous events were known a-priori, then their BOLD responses could be detected conventionally with model-based techniques such as general linear model analysis, but since it is not, then an analysis method that does not need information about the timing of the events is required. PFM has previously been validated as a method for identifying task-induced responses confirmed using electromyography [15,22]. Fig 2B shows that events detected by PFM show similar patterns of activation to those found by standard task induced fMRI. Figs 1 and 2 confirm that short term events contribute to temporal correlations between BOLD signals from different network nodes (as expected since, for instance, any motor action will require multiple nodes of the MN to communicate briefly). Figs 1 and 2 also confirm that network structure is robustly detectable in short time windows [15], suggesting that BOLD measurements of connectivity are driven not only by low frequency oscillations but also by transient fluctuations [13,17–19]. The overlap between the DMN and FPN seen in Fig 2 suggests that there is a dynamic interaction between these networks at the subnetwork level [13] although this interaction is obscured unless functional connectivity is investigated over short time periods. Our results are similar to those from other studies [35,36] that also showed short periods of high correlation contribute to network structure. However in contrast to these previous studies which used point-process analyses, PFM is less subject to confounds from sudden changes in signal due to artefacts (e.g. head jerks) or non-neuronal physiological confounds. This is because PFM assumes that signals that are neuronal in origin must conform to the canonical shape for the haemodynamic response. Some unexpected events still remain (Fig 1A at time t = 148s) where all networks show a coordinated network event and short window (30s) correlation analysis detects the entire grey matter being significantly correlated with a seed taken as any grey matter voxel. These events may relate to sudden global increases in blood flow or oxygenation; they do not relate to motion as determined from the realignment parameters.

Fig 3 builds on previous assessments of dynamic changes in connectivity by showing how the strength of correlation depends on window length used, and how it is affected by a task and by spontaneous events detected by PFM. It shows that as the window length increases, the fraction of windows in which significant activation occurs tends towards unity. When the data are PFM corrected, the whole curve is depressed implying that even long term correlations are driven by events, but the largest change is for window lengths of ~50-60s giving some indication of the length and frequency of events. Coordinated Network Events contributed less than a third of this decrease suggesting that measured functional connectivity is dominated by events that are not detected across *all* network nodes. It was not inevitable that removing voxel wise detected events would reduce correlation: if these events were random or only isolated to specific regions, then their removal might increase correlation by reducing additional variation in the signal.

It is interesting to compare the time-scale of correlation found in fMRI with electrophysiology. Large scale networks detected by MEG bear a striking resemblance to those observed in fMRI [4].We found that nodal events occurred at a rate of ~3 per minute in the MN and FPN, and somewhat faster in the DMN, although these rates may be confounded by the length of the BOLD response, the sparsity constraint used in PFM, the threshold used to define an event in the ATS, and the details of the experimental paradigm since subjects’ attention may drift during the experiment. However, the observed rate concords with results from MEG studies that report signatures of spontaneous brain activity at rest on similar time scales: greatest correlation between MEG signals occurs at <0.25 Hz [37] and maximum coherence between envelopes at ~0.1 Hz [17,18,37,38]. This suggests that temporal characteristics of connectivity measured by different modalities may relate to an inherent rate of occurrence of events.

Fig 5 shows that large scale distributed networks can be decomposed into sets of smaller transiently synchronising sub-networks, each of which involve clusters of voxels contained within multiple nodes of the larger network whose signals are changing in synchrony. These anatomically plausible, transiently synchronising sub-networks are generated on an individual basis, from sparsely populated matrices containing the output from PFM convolved with the canonical HRF (as was used in the deconvolution). The use of the PFM output in this way increases the sensitivity of tICA by reducing the number of confounding temporal components from other sources such as physiological noise, and sensitivity is further enhanced by limiting the analysis to a single network. tICA has been previously applied to raw data [24] but these data sets consisted of many thousands of data points; our approach allows tICA to be used to detect spatially overlapping networks with relatively few temporal points.

The tICA results and the large network structure from combined coordinated network events suggest that the large-scale distributed networks detected over several minutes with fMRI arise from the combined effect of multiple, transiently synchronising sub-networks. In seed-based correlation, the seed timecourse usually collapses signals across many voxels, capturing events related to any components of transiently synchronising sub-networks within the seed. Subsequently correlating this seed timecourse with the rest of the brain will detect all the transiently synchronising sub-networks, which will overlap to define a large, distributed network. Transiently synchronising networks have been described previously [24]; here we considered transient sub-networks within distributed networks based on detected events. tICA also allowed us to find consistent patterns of activity within the network, which will ease the interpretation of the detected BOLD events, simplifying the study of spontaneous brain activity.

The rate of occurrence of spontaneous events and their spatial distribution might provide new markers of behavioural state, and the methods presented here provide an alternative method of studying the brain activity that precedes spontaneous events. We showed that the number of spontaneous events is modulated by some tasks, supporting the findings of other studies [39,40], and that the effect of a task on correlation analysis depends not only on the task and network being considered but also on the length of the correlation window (Fig 3B), which may explain some of the inconsistency in the literature on the effect of tasks on connectivity.

In the DMN and MN, the 2-back task significantly reduced the number of spontaneous events detected, and the fraction of the correlation that could be accounted for by spontaneous events (Figs 3 and 4). In contrast in the FPN the 2-back task reduced the number of spontaneous voxel level events, but did not change the overall network correlation, so that removing events elevated the remaining underlying correlation in the task period compared to the rest period. This suggests a change in the pattern of BOLD signals in the FPN during the task, causing fewer events to be detected/occur, probably because the 2-back task is cognitively demanding. The cognitively demanding 2-back task would have involved multiple parts of the FPN nodes and other brain regions, which may have caused additional effects on other networks. The motor task, requiring fewer mental processes, had less of an effect on background spontaneous events and correlation in the FPN and DMN. The motor task was an extended task which caused a baseline shift in the task-related voxels of the MN, but it did also lead to some increase in voxel-level events in the MN (Fig 4) possibly reflecting either other movements, or variations in task performance; electromyography and video recordings would be required to confirm this. The focal task-related responses only involved a very small cluster of voxels within the network nodes and therefore task related voxels would not have contributed significantly to the nodal events, coordinate network events or average network correlation. Previous work using a focal seed region focused on the task related voxels has reported increased correlation during the task period compared to rest, for short and long time windows [41–43]. We were able to replicate those results if we used a small seed of 27 voxels and this showed a significant increase in correlation (p < 0.05, see supplementary information S3 Fig)

A change in the number of events detected due to the task was found at the voxelwise level but not for nodal or coordinated network events. This may be due to the low number of coordinated network events and hence low statistical power. Alternatively it might be due to the type of event involved. The nodal and coordinated network events typically involve approximately 30% of the network, whereas task related fMRI studies have shown that focal areas of the brain are involved in most tasks. This suggests that nodal or coordinated network events would relate to complicated tasks or stimulation.

These results depend on the success of PFM as a means of detecting spontaneous BOLD events. As currently implemented, PFM uses sparse regression to reduce false positives [23], and is most sensitive to events occurring on the time scale of a single BOLD response, and is less efficient at identifying longer or more frequent responses [44]. Furthermore, the sensitivity of PFM is inevitably limited by the signal to noise ratio of the data. These caveats make it likely that some spontaneous events may have been missed by PFM, and may be contributing to the connectivity detected in null periods (Fig 2) and the strength of the connectivity computed for PFM corrected data (Fig 3). Creating tICA maps from the sparse output from PFM provides a way to use tICA robustly with limited data in the temporal domain. We have used simulations to verify these results but it remains to be seen how the results would be affected by having more voxels with similar timecourses. Finally we have only used 12 subjects to test our methodology; this study needs to be extended to a larger group to provide normative data if these methods are to be used to investigate altered brain function in different conditions.

This work provides novel insights into the mechanisms underlying functional connectivity, suggesting that short events give rise to correlations in signals across recognised networks and that large scale distributed networks are manifestations of smaller transiently synchronising brain networks focused about events.

## Supporting Information

S1 Fig The procedure used to analyse the data. (EPS)

S2 Fig The nodal average timecourses for subject 1 from the motor data in rest period 1. Comparison timecourses for the nodal ATS for Fig 1.
(EPS)

S3 Fig A significant change in correlation coefficient between network nodes from rest period 1 to the task period using focal seeds (27 voxels) for the motor network in the motor data (left) and the fronto-parietal network in the 2-back data. (EPS)

S4 Fig The FSC scores for the motor data showing all four networks. (EPS)

S5 Fig Maps of 10 tICA components for subject 2 depicting the motor network, left fronto-parietal network and default mode network. (EPS)

S6 Fig Maps of 10 tICA components for subject 3 depicting the motor network, left fronto-parietal network and default mode network. (EPS)

S7 Fig Maps of 10 tICA components for subject 4 depicting the motor network, left fronto-parietal network and default mode network. (EPS)

S8 Fig Maps of 10 tICA components for subject 5 depicting the motor network, left fronto-parietal network and default mode network. (EPS)

S9 Fig Maps of 10 tICA components for subject 6 depicting the motor network, left fronto-parietal network and default mode network. (EPS)

S10 Fig Maps of 10 tICA components for subject 7 depicting the motor network, left fronto-parietal network and default mode network. (EPS)

S11 Fig Maps of 10 tICA components for subject 8 depicting the motor network, left fronto-parietal network and default mode network. (EPS)

S12 Fig Maps of 10 tICA components for subject 9 depicting the motor network, left fronto-parietal network and default mode network. (EPS)

S13 Fig Maps of 10 tICA components for subject 10 depicting the motor network, left fronto-parietal network and default mode network. (EPS)

S14 Fig Maps of 10 tICA components for subject 11 depicting the motor network, left fronto-parietal network and default mode network. (EPS)

S15 Fig Maps of 10 tICA components for subject 12 depicting the motor network, left fronto-parietal network and default mode network. (EPS)
